# Poly[dipotassium [(μ_6_-2,2′,2′′,2′′′-{[pyrazine-2,3,5,6-tetra­yltetra­kis­(methyl­ene)]tetra­kis­(sulfanedi­yl)}tetra­acetato)­disilver(I)] 5.2-hydrate]

**DOI:** 10.1107/S2414314622000773

**Published:** 2022-02-01

**Authors:** Jessica Pacifico, Helen Stoeckli-Evans

**Affiliations:** a Institute of Chemistry, University of Neuchâtel, Av. de Bellevax 51, CH-2000 Neuchâtel, Switzerland; b Institute of Physics, University of Neuchâtel, rue Emile-Argand 11, CH-2000 Neuchâtel, Switzerland; University Koblenz-Landau, Germany

**Keywords:** crystal structure, pyrazine, carboxyl­ate, tetra­kis­, silver–potassium–organic framework

## Abstract

The reaction of AgNO_3_ with the ligand 2,2′,2′′,2′′′-{[pyrazine-2,3,5,6-tetra­yltetra­kis­(methyl­ene)]tetra­kis­(sulfanedi­yl)}tetra­acetic acid in the presence of a potassium acetate buffer lead to the formation of a silver(I)–potassium–organic framework, poly[dipotassium [(μ_6_-2,2′,2′′,2′′′-{[pyrazine-2,3,5,6-tetra­yltetra­kis(methyl­ene)]tetra­kis­(sulfanedi­yl)}tetra­acetato)­disilver(I)] 5.2-hydrate], {K_2_[Ag_2_(C_16_H_16_N_2_O_8_S_4_)]·5.2H_2_O}_*n*_, (**I**). The asymmetric unit is composed of half a binuclear silver complex located about a center of symmetry, a potassium cation and 2.6 disordered water mol­ecules. The whole binuclear silver complex is generated by inversion symmetry with the pyrazine ring being located about an inversion centre. The ligand coordinates in a bis-tetra­dentate manner. The binuclear silver complex anions are linked *via* bridging Ag⋯S⋯Ag zigzag bonds, forming a network lying parallel to the *bc* plane. The networks are linked by O_carboxyl­ate_⋯*K* ^+^⋯O_carboxyl­ate_ bridging bonds to form a framework. The disordered water mol­ecules are present near to the K^+^ cations.

## Abstract 2

The reaction of AgNO_3_ with the ligand 2,2′,2′′,2′′′-{[pyrazine-2,3,5,6-tetra­yltetra­kis­(methyl­ene)]tetra­kis­(sulfanedi­yl)}tetra­acetic acid, in the presence of a potassium acetate buffer, lead to the formation of a silver(I)–potassium–organic framework.

## Graphical abstract

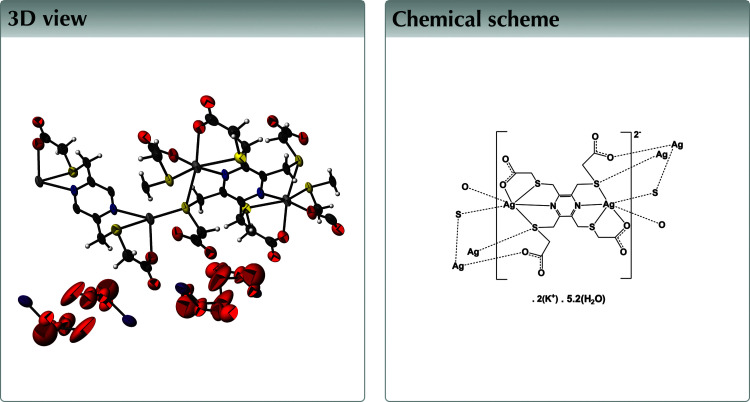

## Structure description

The title ligand, tetra­kis-substituted pyrazine carb­oxy­lic acid, 2,2′,2′′,2′′′-{[pyrazine-2,3,5,6-tetra­yltetra­kis­(methyl­ene)]tetra­kis­(sulfanedi­yl)}tetra­acetic acid (**H_4_L1**), is one of a series of tetra­kis-substituted pyrazine ligands containing N_*x*_S_4_ and N_2_S_4_O_8_ donor atoms (Pacifico, 2003 ▸).

**H_4_L1** is the tetra­acetic acid analogue of 3,3′,3′′,3′′′-{[pyrazine-2,3,5,6-tetra­yltetra­kis(methyl­ene)]tetra­kis­(sulfanedi­yl)}tetra­propionic acid (**H_4_L2**), for which two triclinic polymorphs and two potassium–organic frameworks have been reported (Pacifico & Stoeckli-Evans, 2021*b* ▸ ▸). Reaction of **H_4_L1** with NiCl_2_ lead to the formation of a binuclear complex, {[(H_2_O)_2_Ni_2_(C_16_H_20_N_2_O_8_S_4_)]·7(H_2_O)}, whose crystal structure has been reported (Pacifico & Stoeckli-Evans, 2021*b* ▸).

The reaction of **H_4_L1** (Pacifico & Stoeckli-Evans, 2021*a* ▸) with AgNO_3_ in the presence of a potassium acetate buffer resulted in deprotonation of the ligand and the formation of a heterobimetallic silver(I)–potassium–organic framework (**I**).

**Figure 1:**
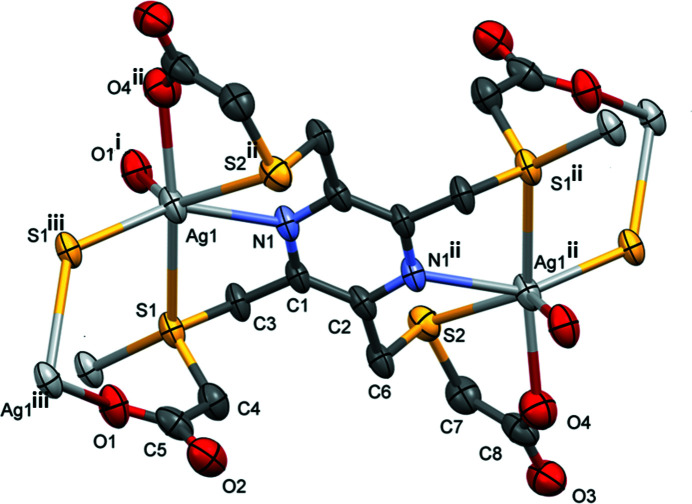
The mol­ecular structure of the silver complex dianion of compound **I**, with atom labelling. Displacement ellipsoids are drawn at the 50% probability level. For clarity, the potassium cation and the disordered water mol­ecules have been omitted. [Symmetry codes: (i) −*x*, *y* + 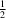 , −*z* + 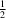 ; (ii) −*x*, −*y* + 1, −*z* + 1; (iii) −*x*, *y* − 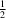 , −*z* + 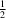 .]

**Figure 2:**
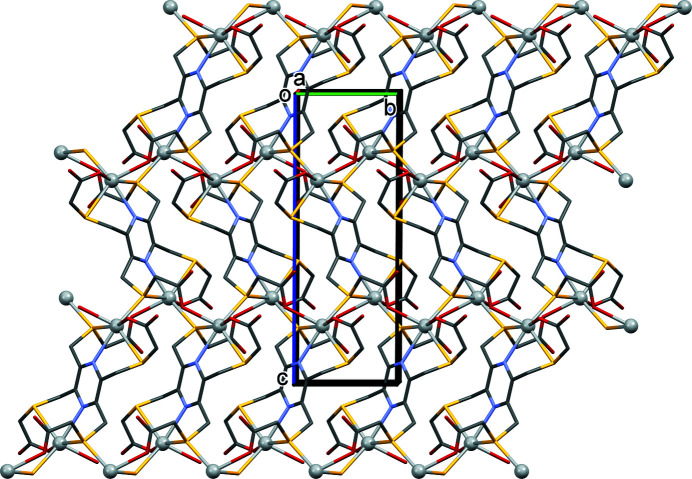
A view along the *a*-axis of the network of the silver complex dianions in compound **I**. The silver atoms are shown as silver balls. For clarity, the potassium ions, the disordered water mol­ecules, and the C-bound H atoms have been omitted.

The asymmetric unit of **I** consists of half a binuclear silver complex, with the ligand coordinating in a bis-tetra­dentate manner (Fig. 1 ▸), a potassium cation and 2.6 disordered water mol­ecules. Selected bond lengths and bond angles involving atom Ag1 are given in Table 1 ▸. The binuclear silver complex anions are linked *via* bridging Ag⋯S⋯Ag zigzag bonds to form a network lying parallel to the *bc* plane (Fig. 2 ▸). The silver ion has a sixfold AgS_3_O_2_N coordination sphere. The bond lengths involving Ag1 fall within the limits observed for the various type of bond when searching the Cambridge Structural Database (CSD, last update September 2021; Groom *et al.*, 2016 ▸). For example, there were over 600 hits for the Ag—N_pyrazine_ bond length that varies from 2.02 to 2.739 Å [mean value 2.321 (89) Å, median 2.304 Å and a skew of 0.866]. In **I** this value is 2.550 (5) Å. For Ag—O_carboxyl­ate_ there were over 2,800 hits with the bond lengths varying from 1.967 to 3.089 Å [mean value 2.377 (147) Å, median 2.352 Å and a skew value of 0.532]. In **I** the Ag—O_carboxyl­ate_ bond lengths are almost equal; 2.470 (5) and 2.466 (6) Å. Finally for the Ag—S(CH2)_2_— bond-length type there were over 1,000 hits with the bond length varying from 2.361 to 3.583 Å [mean value 2.596 (98) Å, median 2.565 Å and a skew value of 1.645]. In **I** the Ag—S(CH2)_2_– bond lengths vary from 2.604 (2) to 2.926 (2) Å, both values involve the bridging atom S1, while distance Ag1—S2^ii^ is 2.824 (2) Å (Table 1 ▸).

The three chelate rings are far from flat, as indicated by the torsion angles given in Table 1 ▸. This is also shown by the mean planes of the chelate rings calculated using *PLATON* (Spek, 2020 ▸): ring Ag1/N1/C2/C3/S1 is twisted on bond S1—C3, ring Ag1/N1/C2^ii^/C6^ii^/S2^ii^ has an envelope conformation with atom S2^ii^ as the flap, and ring Ag^ii^/S2/C7/C8/O4 has an envelope conformation with atom Ag1^ii^ as the flap [symmetry code: (ii) −*x*, −*y* + 1, −*z* + 1].

**Figure 3:**
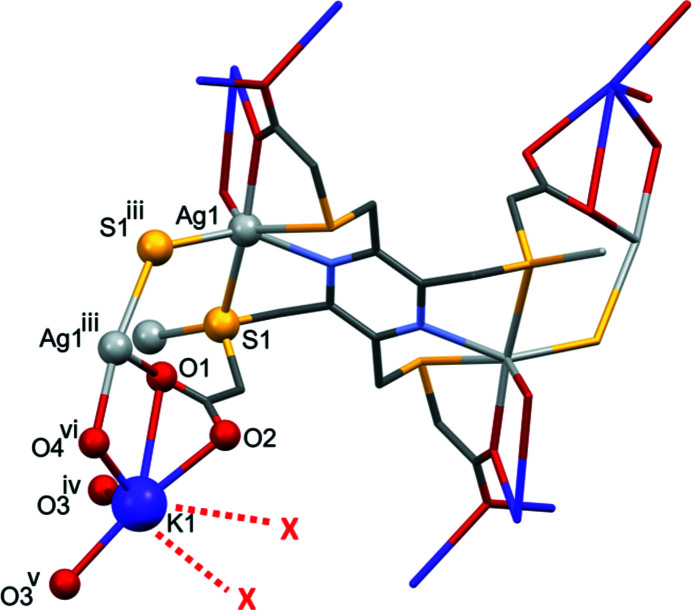
A view of the environment of the potassium cation in compound **I**. [*X*(red) regions of disordered water mol­ecules; symmetry codes: (iii) −*x*, *y* − 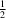 , −*z* + 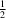 ; (iv) −*x* + 1, −*y* + 1, −*z* + 1; (v) *x*, −*y* + 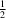 , *z* − 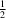 ; (vi) *x*, −*y* + 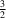 , *z* − 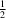 .]

Selected bond lengths and bond angles involving atom K1 are also given in Table 1 ▸. The strongest *K* ^+^⋯O_carboxyl­ate_ bonds lengths vary from 2.608 (6) to 2.751 (6) Å, and there is one weak contact K1⋯O1 at 3.289 (6) Å (Fig. 3 ▸). A search of the CSD for carboxyl­ato–potassium complexes revealed that in the potassium–organic frameworks *catena*-[(μ_4_-3,5,6-tri­carb­oxy­pyrazine-2-carboxyl­ato)potassium] (CSD refcode UBUPAK; Masci *et al.*, 2010 ▸), and *catena*-[(μ-6-carb­oxy­pyridine-2-carboxyl­ato)potassium] (MUMPIW; Li *et al.*, 2020 ▸), the K^+^⋯O bond lengths vary from 2.7951 (11) to 2.8668 (13) Å in UBUPAK and from 2.8197 (14) to 3.0449 (15) Å in MUMPIW. In UBUPAK the K^+^ cation has a coordination number of 8 (KO_8_) and a distorted dodeca­hedral geometry, while in MUMPIW the K^+^ ion has a coordination number of 7 (KO_6_N) and has an edge-sharing penta­gonal anti­prism geometry. In **I**, the stronger K⋯O bond lengths are shorter and, owing to the presence of the disordered water mol­ecules, it is not clear what the K^+^ ion coordination number or geometry are.

**Figure 4:**
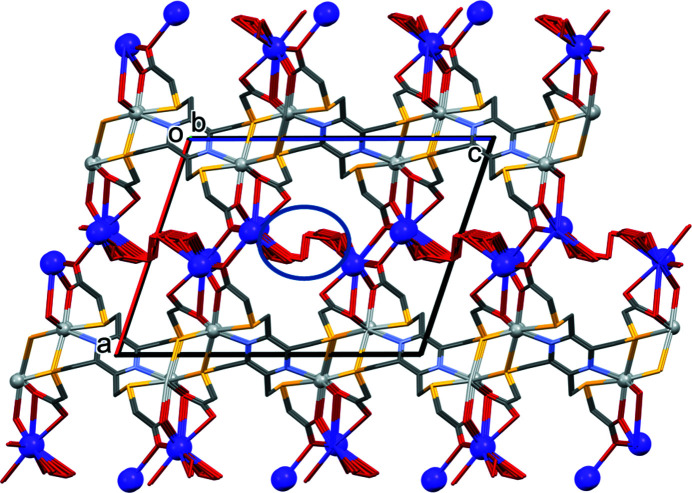
A view along the *b*-axis of the crystal packing of compound **I**. The silver atoms are shown as small silver balls and the potassium ions as large purple balls. The blue ellipse indicates the region occupied by the disordered water mol­ecules. For clarity, the C-bound H atoms have been omitted.

In the crystal of **I**, the networks of the binuclear silver complex anions are linked by the bridging O_carboxyl­ate_⋯*K* ^+^⋯O_carboxyl­ate_ bonds to form a framework (Fig. 4 ▸; Table 1 ▸). The disordered water mol­ecules are present near to the K^+^ cations.

## Synthesis and crystallization

The synthesis of the ligand **H_4_L1** has been described (Pacifico & Stoeckli-Evans, 2021*a* ▸).

**Synthesis of poly{(** ***μ*** **-2,2′,2′′,2′′′-{[pyrazine-2,3,5,6-tetra­yltetra­kis­(methyl­ene)] tetra­kis­(sulfanedi­yl)}tetra­acetato)}-bis­[silver(I)]-bis­[potassium] 5.2(hydrate)} (I):**

AgNO_3_ (20.5 mg, 0.121 mmol, 2 eq) and **H_4_L1** (30 mg, 0.060 mmol, 1 eq) were mixed in 20 ml of a 1*M* potassium acetate buffer solution. The mixture was left at 323 K under stirring and nitro­gen conditions for 1 h. The mixture was then filtered and left to evaporate in air for six weeks, yielding yellow rod-like crystals of compound **I** (m.p. 553 K decomposition).

Analysis for C_16_H_16_Ag_2_N_2_O_8_S_4_, K_2_, 5.2(H_2_O), *M*_w_ = 880.175 g mol^−1^: Calculated (%): C 21.88, H 2.99, N 3.18. Found (%): C 23.03, H 2.91, N 3.03. The small deviation is probably due to the loss of water mol­ecules of crystallization.

ESI–MS: unstable under mass spectroscopy experimental conditions.

IR (KBr disc, cm^−1^) ν: 3401(*s*), 2938(*m*), 1599(*s*), 1385(*s*), 1223(*m*).

## Refinement

Crystal data, data collection and structure refinement details are summarized in Table 2 ▸. The occupancy factors for the disordered water mol­ecules were initially freely refined and then fixed at rounded values; the final total is 5.2(H_2_O). It was not possible to locate the H atoms of the disordered water mol­ecules of crystallization. The residual electron density peaks of 1.14 and −1.10 eÅ^3^ are at distances of 0.96 and 0.91 Å, respectively, from atom Ag1.

## Supplementary Material

Crystal structure: contains datablock(s) I, Global. DOI: 10.1107/S2414314622000773/im4015sup1.cif

Structure factors: contains datablock(s) I. DOI: 10.1107/S2414314622000773/im4015Isup2.hkl

CCDC reference: 2143798

Additional supporting information:  crystallographic information; 3D view; checkCIF report

## Figures and Tables

**Table 1:** Selected geometric parameters (Å, °)

Ag1—N1	2.550 (5)	K1—O1	3.289 (6)
Ag1—O1^i^	2.470 (5)	K1—O2	2.729 (6)
Ag1—O4^ii^	2.466 (6)	K1—O3^iv^	2.724 (6)
Ag1—S1	2.926 (2)	K1—O3^v^	2.751 (6)
Ag1—S1^iii^	2.604 (2)	K1—O4^vi^	2.608 (6)
Ag1—S2^ii^	2.824 (2)		
			
O4^ii^—Ag1—O1^i^	90.01 (19)	S1^iii^—Ag1—S1	87.60 (4)
O4^ii^—Ag1—N1	110.25 (18)	S2^ii^—Ag1—S1	122.15 (6)
O1^i^—Ag1—N1	84.78 (17)	Ag1^i^—S1—Ag1	129.26 (7)
O4^ii^—Ag1—S1^iii^	96.08 (14)	O4^vi^—K1—O3^iv^	94.50 (18)
O1^i^—Ag1—S1^iii^	108.15 (12)	O4^vi^—K1—O2	89.60 (19)
N1—Ag1—S1^iii^	150.87 (13)	O3^iv^—K1—O2	170.9 (2)
O4^ii^—Ag1—S2^ii^	69.66 (14)	O4^vi^—K1—O3^v^	116.0 (2)
O1^i^—Ag1—S2^ii^	138.52 (12)	O3^iv^—K1—O3^v^	86.76 (14)
N1—Ag1—S2^ii^	70.23 (12)	O2—K1—O3^v^	98.72 (18)
S1^iii^—Ag1—S2^ii^	109.63 (5)	O4^vi^—K1—O1	71.53 (16)
O4^ii^—Ag1—S1	165.62 (14)	O3^iv^—K1—O1	146.36 (18)
O1^i^—Ag1—S1	75.65 (14)	O2—K1—O1	42.72 (15)
N1—Ag1—S1	70.04 (12)	O3^v^—K1—O1	73.37 (15)
			
N1—C1—C3—S1	−61.4 (7)	S2—C7—C8—O4	1.5 (10)
N1^ii^—C2—C6—S2	−70.4 (7)		

Symmetry codes: (i) 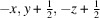 ; (ii) 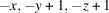 ; (iii) 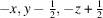 ; (iv) 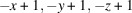 ; (v) 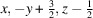 ; (vi) 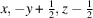 .

**Table 2:** Experimental details

Crystal data	
Chemical formula	K_2_[Ag_2_(C_16_H_16_N_2_O_8_S_4_)]5.2H_2_O
*M*_r_	880.17
Crystal system, space group	Monoclinic, *P*2_1_/*c*
Temperature (K)	153
*a*, *b*, *c* (Å)	13.386 (3), 6.0085 (7), 17.843 (3)
β (°)	108.657 (15)
*V* (Å^3^)	1359.7 (4)
*Z*	2
Radiation type	Mo *K*α
μ (mm^−1^)	2.12
Crystal size (mm)	0.24 × 0.13 × 0.05

Data collection	
Diffractometer	Stoe *IPDS* 2
Absorption correction	Multi-scan (*MULABS*; Spek, 2020 ▸)
*T*_min_, *T*_max_	0.611, 1.000
No. of measured, independent and observed [*I* > 2σ(*I*)] reflections	9305, 2316, 2088
*R*_int_	0.043
(sin θ/λ)_max_ (Å^−1^)	0.591

Refinement	
*R*[*F* ^2^ > 2σ(*F* ^2^)], *wR*(*F* ^2^), *S*	0.048, 0.114, 1.17
No. of reflections	2316
No. of parameters	218
H-atom treatment	H-atom parameters constrained
Δρ_max_, Δρ_min_ (e Å^−3^)	1.14, −1.10

Computer programs: *X-AREA* (Stoe & Cie, 2002 ▸), *X-RED32* (Stoe & Cie, 2002 ▸), *SHELXS97* (Sheldrick, 2008 ▸), *PLATON* (Spek, 2020 ▸) and *Mercury* (Macrae *et al.*, 2020 ▸), *SHELXL2018/3* (Sheldrick, 2015 ▸), *PLATON* (Spek, 2020 ▸) and *publCIF* (Westrip, 2010 ▸).

## full crystallographic data

### Crystal data

**Table:**

K_2_[Ag_2_(C_16_H_16_N_2_O_8_S_4_)]5.2H_2_O	*F*(000) = 852
*M**_r_* = 880.17	*D*_x_ = 2.097 Mg m^−^^3^
Monoclinic, *P*2_1_/*c*	Mo *K*α radiation, λ = 0.71073 Å
*a* = 13.386 (3) Å	Cell parameters from 17882 reflections
*b* = 6.0085 (7) Å	θ = 1.6–24.9°
*c* = 17.843 (3) Å	µ = 2.12 mm^−^^1^
β = 108.657 (15)°	*T* = 153 K
*V* = 1359.7 (4) Å^3^	Rod, yellow
*Z* = 2	0.24 × 0.13 × 0.05 mm

### Data collection

**Table:**

Stoe IPDS 2 diffractometer	2316 independent reflections
Radiation source: fine-focus sealed tube	2088 reflections with *I* > 2σ(*I*)
Plane graphite monochromator	*R*_int_ = 0.043
φ + ω scans	θ_max_ = 24.8°, θ_min_ = 2.4°
Absorption correction: multi-scan (*MULABS*; Spek, 2020)	*h* = −15→15
*T*_min_ = 0.611, *T*_max_ = 1.000	*k* = −7→6
9305 measured reflections	*l* = −20→21

### Refinement

**Table:**

Refinement on *F*^2^	Secondary atom site location: difference Fourier map
Least-squares matrix: full	Hydrogen site location: inferred from neighbouring sites
*R*[*F*^2^ > 2σ(*F*^2^)] = 0.048	H-atom parameters constrained
*wR*(*F*^2^) = 0.114	*w* = 1/[σ^2^(*F*_o_^2^) + (0.0335*P*)^2^ + 8.3123*P*] where *P* = (*F*_o_^2^ + 2*F*_c_^2^)/3
*S* = 1.17	(Δ/σ)_max_ < 0.001
2316 reflections	Δρ_max_ = 1.14 e Å^−^^3^
218 parameters	Δρ_min_ = −1.10 e Å^−^^3^
0 restraints	Extinction correction: (SHELXL2018/3; Sheldrick, 2015), Fc^*^=kFc[1+0.001xFc^2^λ^3^/sin(2θ)]^-1/4^
Primary atom site location: structure-invariant direct methods	Extinction coefficient: 0.0058 (8)

### Special details

**Table:**

Geometry. All esds (except the esd in the dihedral angle between two l.s. planes) are estimated using the full covariance matrix. The cell esds are taken into account individually in the estimation of esds in distances, angles and torsion angles; correlations between esds in cell parameters are only used when they are defined by crystal symmetry. An approximate (isotropic) treatment of cell esds is used for estimating esds involving l.s. planes.
Refinement. The C-bound H atoms were included in calculated positions and treated as riding on their parent C atom: C—H = 0.99 Å with *U*_iso_(H) = 1.2*U*_eq_(C).

### Fractional atomic coordinates and isotropic or equivalent isotropic displacement parameters (Å^2^)

**Table:**

	*x*	*y*	*z*	*U*_iso_*/*U*_eq_	Occ. (<1)
Ag1	−0.11736 (5)	0.22235 (9)	0.29645 (3)	0.0422 (2)	
K1	0.42397 (15)	−0.0830 (3)	0.31383 (13)	0.0662 (6)	
S1	0.07736 (14)	0.4594 (3)	0.30640 (9)	0.0383 (4)	
S2	0.13892 (15)	1.0313 (3)	0.57635 (11)	0.0456 (5)	
O1	0.1777 (5)	0.0832 (8)	0.2686 (3)	0.0534 (13)	
O2	0.2813 (5)	−0.0081 (9)	0.3913 (3)	0.0602 (15)	
O3	0.4123 (4)	1.1764 (10)	0.7406 (4)	0.0625 (15)	
O4	0.3022 (5)	0.8947 (10)	0.7311 (3)	0.0617 (15)	
N1	−0.0564 (4)	0.4506 (9)	0.4235 (3)	0.0363 (13)	
C1	0.0288 (5)	0.5773 (10)	0.4399 (3)	0.0335 (14)	
C2	0.0881 (5)	0.6285 (10)	0.5180 (4)	0.0367 (15)	
C3	0.0533 (6)	0.6731 (11)	0.3695 (4)	0.0392 (15)	
H3A	0.116280	0.769845	0.388415	0.047*	
H3B	−0.006596	0.766385	0.338485	0.047*	
C4	0.1990 (6)	0.3419 (12)	0.3732 (4)	0.0486 (18)	
H4A	0.258373	0.444967	0.377860	0.058*	
H4B	0.192240	0.321755	0.426414	0.058*	
C5	0.2207 (6)	0.1200 (12)	0.3415 (5)	0.0478 (18)	
C6	0.1824 (6)	0.7758 (11)	0.5427 (4)	0.0397 (15)	
H6A	0.208510	0.805366	0.497629	0.048*	
H6B	0.239747	0.706121	0.585903	0.048*	
C7	0.2611 (6)	1.1711 (12)	0.6282 (5)	0.0508 (18)	
H7A	0.303971	1.179423	0.592207	0.061*	
H7B	0.244080	1.325716	0.638995	0.061*	
C8	0.3293 (6)	1.0693 (13)	0.7059 (5)	0.052 (2)	
O1W	0.479 (2)	0.483 (5)	0.3773 (16)	0.072 (7)	0.3
O2W	0.4522 (18)	0.660 (4)	0.0890 (13)	0.071 (6)	0.3
O3W	0.4365 (15)	0.538 (4)	0.0344 (9)	0.123 (7)	0.5
O4W	0.4421 (18)	0.285 (3)	0.0195 (13)	0.105 (7)	0.4
O5W	0.4629 (16)	0.844 (4)	0.0802 (13)	0.057 (5)	0.3
O6W	0.451 (3)	0.951 (7)	0.115 (2)	0.198 (17)	0.5
O7W	0.556 (4)	0.047 (9)	0.009 (3)	0.23 (3)	0.3

### Atomic displacement parameters (Å^2^)

**Table:**

	*U*^11^	*U*^22^	*U*^33^	*U*^12^	*U*^13^	*U*^23^
Ag1	0.0620 (4)	0.0363 (3)	0.0386 (3)	−0.0055 (2)	0.0305 (3)	−0.0060 (2)
K1	0.0607 (11)	0.0602 (12)	0.0891 (14)	0.0039 (9)	0.0400 (10)	0.0048 (10)
S1	0.0579 (11)	0.0325 (8)	0.0332 (8)	−0.0005 (7)	0.0266 (8)	−0.0006 (7)
S2	0.0552 (11)	0.0347 (9)	0.0531 (11)	−0.0041 (8)	0.0261 (9)	−0.0089 (8)
O1	0.082 (4)	0.037 (3)	0.050 (3)	0.003 (3)	0.034 (3)	−0.002 (2)
O2	0.069 (4)	0.053 (3)	0.067 (3)	0.011 (3)	0.033 (3)	0.015 (3)
O3	0.059 (4)	0.059 (4)	0.075 (4)	−0.012 (3)	0.030 (3)	−0.018 (3)
O4	0.070 (4)	0.057 (3)	0.064 (4)	−0.007 (3)	0.030 (3)	0.005 (3)
N1	0.057 (4)	0.031 (3)	0.028 (3)	−0.002 (3)	0.024 (2)	−0.001 (2)
C1	0.050 (4)	0.028 (3)	0.030 (3)	0.000 (3)	0.025 (3)	−0.004 (3)
C2	0.055 (4)	0.027 (3)	0.039 (3)	0.001 (3)	0.031 (3)	−0.001 (3)
C3	0.064 (4)	0.032 (3)	0.030 (3)	−0.003 (3)	0.028 (3)	−0.001 (3)
C4	0.063 (5)	0.045 (4)	0.045 (4)	0.002 (4)	0.028 (4)	0.004 (3)
C5	0.059 (5)	0.034 (4)	0.065 (5)	0.003 (3)	0.040 (4)	0.010 (4)
C6	0.059 (4)	0.032 (3)	0.039 (3)	−0.005 (3)	0.030 (3)	−0.007 (3)
C7	0.065 (5)	0.036 (4)	0.058 (5)	−0.010 (3)	0.030 (4)	−0.005 (3)
C8	0.057 (5)	0.045 (4)	0.069 (5)	−0.013 (4)	0.039 (4)	−0.019 (4)
O1W	0.081 (17)	0.052 (12)	0.066 (14)	0.000 (13)	0.000 (13)	0.017 (11)
O2W	0.084 (15)	0.055 (14)	0.060 (13)	−0.001 (11)	0.003 (11)	−0.004 (11)
O3W	0.123 (15)	0.18 (2)	0.065 (10)	0.040 (14)	0.024 (9)	−0.005 (12)
O4W	0.113 (17)	0.077 (13)	0.111 (16)	0.001 (12)	0.016 (13)	0.011 (12)
O5W	0.044 (11)	0.043 (12)	0.062 (13)	0.006 (9)	−0.015 (9)	0.000 (10)
O6W	0.22 (4)	0.16 (3)	0.21 (3)	0.05 (3)	0.06 (3)	−0.01 (3)
O7W	0.21 (5)	0.21 (5)	0.16 (4)	0.08 (5)	−0.09 (4)	−0.02 (4)

### Geometric parameters (Å, º)

**Table:**

Ag1—N1	2.550 (5)	O4—C8	1.241 (9)
Ag1—O1^i^	2.470 (5)	N1—C1	1.324 (8)
Ag1—O4^ii^	2.466 (6)	N1—C2^ii^	1.334 (8)
Ag1—S1	2.926 (2)	C1—C2	1.399 (9)
Ag1—S1^iii^	2.604 (2)	C1—C3	1.509 (8)
Ag1—S2^ii^	2.824 (2)	C2—C6	1.489 (10)
Ag1—K1^i^	4.113 (2)	C3—H3A	0.9900
K1—O2W^iv^	2.46 (2)	C3—H3B	0.9900
K1—O1	3.289 (6)	C4—C5	1.512 (10)
K1—O2	2.729 (6)	C4—H4A	0.9900
K1—O3^v^	2.724 (6)	C4—H4B	0.9900
K1—O3^vi^	2.751 (6)	C6—H6A	0.9900
K1—O4^vii^	2.608 (6)	C6—H6B	0.9900
K1—O1W^viii^	2.84 (3)	C7—C8	1.523 (12)
K1—O3W^iv^	2.848 (17)	C7—H7A	0.9900
K1—O4W^iv^	3.05 (2)	C7—H7B	0.9900
K1—C5	3.162 (7)	O1W—O6W^iv^	0.93 (4)
K1—O5W^iv^	3.26 (2)	O1W—O5W^iv^	1.22 (4)
K1—O6W^ix^	3.30 (4)	O2W—O5W	1.13 (3)
S1—C3	1.803 (6)	O2W—O3W	1.19 (3)
S1—C4	1.824 (8)	O2W—O6W	1.81 (4)
S2—C7	1.808 (8)	O3W—O4W	1.55 (3)
S2—C6	1.811 (7)	O5W—O6W	0.94 (4)
O1—C5	1.263 (9)	O5W—O7W^x^	1.66 (6)
O2—C5	1.256 (9)	O7W—O7W^xi^	1.53 (11)
O3—C8	1.262 (9)		
			
O4^ii^—Ag1—O1^i^	90.01 (19)	C7—S2—C6	103.3 (4)
O4^ii^—Ag1—N1	110.25 (18)	C7—S2—Ag1^ii^	98.5 (3)
O1^i^—Ag1—N1	84.78 (17)	C6—S2—Ag1^ii^	86.3 (2)
O4^ii^—Ag1—S1^iii^	96.08 (14)	C5—O1—Ag1^iii^	127.6 (5)
O1^i^—Ag1—S1^iii^	108.15 (12)	C5—O1—K1	73.1 (4)
N1—Ag1—S1^iii^	150.87 (13)	Ag1^iii^—O1—K1	90.02 (15)
O4^ii^—Ag1—S2^ii^	69.66 (14)	C5—O2—K1	98.2 (4)
O1^i^—Ag1—S2^ii^	138.52 (12)	C8—O3—K1^v^	113.7 (5)
N1—Ag1—S2^ii^	70.23 (12)	C8—O3—K1^xii^	126.4 (5)
S1^iii^—Ag1—S2^ii^	109.63 (5)	K1^v^—O3—K1^xii^	115.0 (2)
O4^ii^—Ag1—S1	165.62 (14)	C8—O4—Ag1^ii^	124.0 (6)
O1^i^—Ag1—S1	75.65 (14)	C8—O4—K1^xiii^	127.6 (5)
N1—Ag1—S1	70.04 (12)	Ag1^ii^—O4—K1^xiii^	108.3 (2)
S1^iii^—Ag1—S1	87.60 (4)	C1—N1—C2^ii^	119.9 (6)
S2^ii^—Ag1—S1	122.15 (6)	C1—N1—Ag1	120.9 (4)
O4^ii^—Ag1—K1^i^	37.02 (14)	C2^ii^—N1—Ag1	114.4 (4)
O1^i^—Ag1—K1^i^	53.08 (13)	N1—C1—C2	121.4 (5)
N1—Ag1—K1^i^	105.00 (13)	N1—C1—C3	115.9 (6)
S1^iii^—Ag1—K1^i^	103.54 (5)	C2—C1—C3	122.7 (6)
S2^ii^—Ag1—K1^i^	101.25 (5)	N1^ii^—C2—C1	118.7 (6)
S1—Ag1—K1^i^	128.60 (5)	N1^ii^—C2—C6	115.6 (6)
Ag1^i^—S1—Ag1	129.26 (7)	C1—C2—C6	125.6 (5)
O2W^iv^—K1—O4^vii^	169.6 (6)	C1—C3—S1	112.2 (4)
O2W^iv^—K1—O3^v^	86.3 (6)	C1—C3—H3A	109.2
O4^vii^—K1—O3^v^	94.50 (18)	S1—C3—H3A	109.2
O2W^iv^—K1—O2	88.2 (6)	C1—C3—H3B	109.2
O4^vii^—K1—O2	89.60 (19)	S1—C3—H3B	109.2
O3^v^—K1—O2	170.9 (2)	H3A—C3—H3B	107.9
O2W^iv^—K1—O3^vi^	74.4 (6)	C5—C4—S1	109.6 (5)
O4^vii^—K1—O3^vi^	116.0 (2)	C5—C4—H4A	109.7
O3^v^—K1—O3^vi^	86.76 (14)	S1—C4—H4A	109.7
O2—K1—O3^vi^	98.72 (18)	C5—C4—H4B	109.7
O2W^iv^—K1—O1W^viii^	103.5 (8)	S1—C4—H4B	109.7
O4^vii^—K1—O1W^viii^	66.6 (6)	H4A—C4—H4B	108.2
O3^v^—K1—O1W^viii^	79.4 (6)	O2—C5—O1	126.8 (7)
O2—K1—O1W^viii^	94.8 (6)	O2—C5—C4	115.7 (7)
O3^vi^—K1—O1W^viii^	166.2 (6)	O1—C5—C4	117.4 (7)
O2W^iv^—K1—O3W^iv^	24.4 (6)	O2—C5—K1	58.7 (4)
O4^vii^—K1—O3W^iv^	145.2 (5)	O1—C5—K1	84.4 (4)
O3^v^—K1—O3W^iv^	91.9 (4)	C4—C5—K1	132.6 (5)
O2—K1—O3W^iv^	80.2 (4)	C2—C6—S2	105.7 (5)
O3^vi^—K1—O3W^iv^	98.4 (5)	C2—C6—H6A	110.6
O1W^viii^—K1—O3W^iv^	81.2 (8)	S2—C6—H6A	110.6
O2W^iv^—K1—O4W^iv^	54.0 (7)	C2—C6—H6B	110.6
O4^vii^—K1—O4W^iv^	115.6 (4)	S2—C6—H6B	110.6
O3^v^—K1—O4W^iv^	90.3 (5)	H6A—C6—H6B	108.7
O2—K1—O4W^iv^	80.6 (5)	C8—C7—S2	117.4 (5)
O3^vi^—K1—O4W^iv^	128.4 (4)	C8—C7—H7A	108.0
O1W^viii^—K1—O4W^iv^	51.4 (7)	S2—C7—H7A	108.0
O3W^iv^—K1—O4W^iv^	30.2 (6)	C8—C7—H7B	108.0
O2W^iv^—K1—C5	94.6 (6)	S2—C7—H7B	108.0
O4^vii^—K1—C5	87.3 (2)	H7A—C7—H7B	107.2
O3^v^—K1—C5	165.0 (2)	O4—C8—O3	124.6 (9)
O2—K1—C5	23.16 (17)	O4—C8—C7	120.7 (7)
O3^vi^—K1—C5	79.11 (18)	O3—C8—C7	114.8 (7)
O1W^viii^—K1—C5	114.7 (6)	O4—C8—K1^v^	117.4 (5)
O3W^iv^—K1—C5	95.1 (5)	O3—C8—K1^v^	46.6 (4)
O4W^iv^—K1—C5	102.4 (5)	C7—C8—K1^v^	102.3 (4)
O2W^iv^—K1—O5W^iv^	16.3 (6)	O4—C8—K1^xiii^	36.1 (4)
O4^vii^—K1—O5W^iv^	169.7 (4)	O3—C8—K1^xiii^	92.8 (5)
O3^v^—K1—O5W^iv^	95.3 (4)	C7—C8—K1^xiii^	147.2 (5)
O2—K1—O5W^iv^	81.2 (5)	K1^v^—C8—K1^xiii^	83.44 (19)
O3^vi^—K1—O5W^iv^	61.4 (4)	O6W^iv^—O1W—O5W^iv^	50 (3)
O1W^viii^—K1—O5W^iv^	118.5 (7)	O6W^iv^—O1W—K1^xiv^	112 (4)
O3W^iv^—K1—O5W^iv^	37.5 (6)	O5W^iv^—O1W—K1^xiv^	155 (2)
O4W^iv^—K1—O5W^iv^	67.6 (5)	O5W—O2W—O3W	119 (3)
C5—K1—O5W^iv^	82.5 (4)	O5W—O2W—O6W	27 (2)
O2W^iv^—K1—O1	112.9 (6)	O3W—O2W—O6W	143 (2)
O4^vii^—K1—O1	71.53 (16)	O5W—O2W—K1^xv^	126.2 (17)
O3^v^—K1—O1	146.36 (18)	O3W—O2W—K1^xv^	96.4 (16)
O2—K1—O1	42.72 (15)	O6W—O2W—K1^xv^	117.0 (16)
O3^vi^—K1—O1	73.37 (15)	O2W—O3W—O4W	138 (2)
O1W^viii^—K1—O1	119.2 (6)	O2W—O3W—K1^xv^	59.1 (13)
O3W^iv^—K1—O1	117.2 (5)	O4W—O3W—K1^xv^	82.0 (11)
O4W^iv^—K1—O1	123.3 (5)	O3W—O4W—K1^xv^	67.8 (10)
C5—K1—O1	22.47 (17)	O6W—O5W—O2W	121 (4)
O5W^iv^—K1—O1	98.4 (4)	O6W—O5W—O1W^xv^	49 (3)
O2W^iv^—K1—O6W^ix^	95.0 (8)	O2W—O5W—O1W^xv^	131 (2)
O4^vii^—K1—O6W^ix^	76.0 (7)	O6W—O5W—O7W^x^	111 (4)
O3^v^—K1—O6W^ix^	66.1 (7)	O2W—O5W—O7W^x^	122 (3)
O2—K1—O6W^ix^	107.3 (7)	O1W^xv^—O5W—O7W^x^	101 (3)
O3^vi^—K1—O6W^ix^	151.6 (7)	O6W—O5W—K1^xv^	107 (3)
O1W^viii^—K1—O6W^ix^	15.1 (8)	O2W—O5W—K1^xv^	37.5 (13)
O3W^iv^—K1—O6W^ix^	75.6 (9)	O1W^xv^—O5W—K1^xv^	95.3 (16)
O4W^iv^—K1—O6W^ix^	48.4 (8)	O7W^x^—O5W—K1^xv^	140 (2)
C5—K1—O6W^ix^	128.6 (7)	O1W^xv^—O6W—O5W	82 (4)
O5W^iv^—K1—O6W^ix^	111.1 (7)	O1W^xv^—O6W—O2W	98 (4)
O1—K1—O6W^ix^	134.3 (7)	O5W—O6W—O2W	32 (2)
C3—S1—C4	99.7 (3)	O1W^xv^—O6W—K1^xvi^	53 (3)
C3—S1—Ag1^i^	97.3 (2)	O5W—O6W—K1^xvi^	131 (4)
C4—S1—Ag1^i^	110.7 (2)	O2W—O6W—K1^xvi^	151 (2)
C3—S1—Ag1	92.9 (2)	O7W^xi^—O7W—O5W^x^	96 (3)
C4—S1—Ag1	116.3 (3)		
			
C2^ii^—N1—C1—C2	0.9 (10)	K1^xii^—O3—C8—C7	70.3 (8)
Ag1—N1—C1—C2	−153.3 (5)	K1^xii^—O3—C8—K1^v^	153.8 (8)
C2^ii^—N1—C1—C3	−175.4 (6)	K1^v^—O3—C8—K1^xiii^	78.2 (4)
Ag1—N1—C1—C3	30.4 (7)	K1^xii^—O3—C8—K1^xiii^	−128.0 (4)
N1—C1—C2—N1^ii^	−0.9 (10)	S2—C7—C8—O4	1.5 (10)
C3—C1—C2—N1^ii^	175.2 (6)	S2—C7—C8—O3	−178.3 (5)
N1—C1—C2—C6	−177.5 (6)	S2—C7—C8—K1^v^	134.0 (4)
C3—C1—C2—C6	−1.4 (10)	S2—C7—C8—K1^xiii^	37.0 (11)
N1—C1—C3—S1	−61.4 (7)	O5W—O2W—O3W—O4W	162 (3)
C2—C1—C3—S1	122.3 (6)	O6W—O2W—O3W—O4W	179 (3)
C4—S1—C3—C1	−66.2 (6)	K1^xv^—O2W—O3W—O4W	24 (3)
Ag1^i^—S1—C3—C1	−178.7 (5)	O5W—O2W—O3W—K1^xv^	138 (3)
Ag1—S1—C3—C1	51.1 (5)	O6W—O2W—O3W—K1^xv^	155 (4)
C3—S1—C4—C5	165.4 (5)	O2W—O3W—O4W—K1^xv^	−21 (3)
Ag1^i^—S1—C4—C5	−93.0 (5)	O3W—O2W—O5W—O6W	157 (4)
Ag1—S1—C4—C5	67.3 (5)	K1^xv^—O2W—O5W—O6W	−78 (4)
K1—O2—C5—O1	53.3 (8)	O3W—O2W—O5W—O1W^xv^	−143 (3)
K1—O2—C5—C4	−125.8 (5)	O6W—O2W—O5W—O1W^xv^	60 (4)
Ag1^iii^—O1—C5—O2	33.0 (11)	K1^xv^—O2W—O5W—O1W^xv^	−18 (5)
K1—O1—C5—O2	−43.5 (7)	O3W—O2W—O5W—O7W^x^	7 (4)
Ag1^iii^—O1—C5—C4	−147.9 (5)	O6W—O2W—O5W—O7W^x^	−150 (6)
K1—O1—C5—C4	135.6 (6)	K1^xv^—O2W—O5W—O7W^x^	132 (3)
Ag1^iii^—O1—C5—K1	76.5 (5)	O3W—O2W—O5W—K1^xv^	−125 (4)
S1—C4—C5—O2	−159.1 (5)	O6W—O2W—O5W—K1^xv^	78 (4)
S1—C4—C5—O1	21.6 (8)	O2W—O5W—O6W—O1W^xv^	120 (4)
S1—C4—C5—K1	130.8 (5)	O7W^x^—O5W—O6W—O1W^xv^	−87 (4)
N1^ii^—C2—C6—S2	−70.4 (7)	K1^xv^—O5W—O6W—O1W^xv^	81 (4)
C1—C2—C6—S2	106.2 (6)	O1W^xv^—O5W—O6W—O2W	−120 (4)
C7—S2—C6—C2	165.8 (5)	O7W^x^—O5W—O6W—O2W	153 (5)
Ag1^ii^—S2—C6—C2	68.0 (4)	K1^xv^—O5W—O6W—O2W	−39 (2)
C6—S2—C7—C8	−69.2 (6)	O2W—O5W—O6W—K1^xvi^	142 (3)
Ag1^ii^—S2—C7—C8	18.9 (6)	O1W^xv^—O5W—O6W—K1^xvi^	22 (2)
Ag1^ii^—O4—C8—O3	150.8 (6)	O7W^x^—O5W—O6W—K1^xvi^	−65 (5)
K1^xiii^—O4—C8—O3	−32.5 (10)	K1^xv^—O5W—O6W—K1^xvi^	103 (4)
Ag1^ii^—O4—C8—C7	−29.0 (9)	O5W—O2W—O6W—O1W^xv^	−60 (5)
K1^xiii^—O4—C8—C7	147.7 (6)	O3W—O2W—O6W—O1W^xv^	−94 (5)
Ag1^ii^—O4—C8—K1^v^	−154.8 (3)	K1^xv^—O2W—O6W—O1W^xv^	58 (5)
K1^xiii^—O4—C8—K1^v^	21.9 (8)	O3W—O2W—O6W—O5W	−34 (6)
Ag1^ii^—O4—C8—K1^xiii^	−176.7 (9)	K1^xv^—O2W—O6W—O5W	118 (4)
K1^v^—O3—C8—O4	96.7 (8)	O5W—O2W—O6W—K1^xvi^	−72 (5)
K1^xii^—O3—C8—O4	−109.5 (8)	O3W—O2W—O6W—K1^xvi^	−106 (5)
K1^v^—O3—C8—C7	−83.5 (7)	K1^xv^—O2W—O6W—K1^xvi^	45 (5)

Symmetry codes: (i) −*x*, *y*+1/2, −*z*+1/2; (ii) −*x*, −*y*+1, −*z*+1; (iii) −*x*, *y*−1/2, −*z*+1/2; (iv) −*x*+1, *y*−1/2, −*z*+1/2; (v) −*x*+1, −*y*+1, −*z*+1; (vi) *x*, −*y*+3/2, *z*−1/2; (vii) *x*, −*y*+1/2, *z*−1/2; (viii) *x*, *y*−1, *z*; (ix) −*x*+1, *y*−3/2, −*z*+1/2; (x) −*x*+1, −*y*+1, −*z*; (xi) −*x*+1, −*y*, −*z*; (xii) *x*, −*y*+3/2, *z*+1/2; (xiii) *x*, −*y*+1/2, *z*+1/2; (xiv) *x*, *y*+1, *z*; (xv) −*x*+1, *y*+1/2, −*z*+1/2; (xvi) −*x*+1, *y*+3/2, −*z*+1/2.
